# Molecular Responses to Avian Reovirus Inoculation In Vitro

**DOI:** 10.3390/v17111489

**Published:** 2025-11-10

**Authors:** Zubair Khalid, Ruediger Hauck

**Affiliations:** 1 Department of Pathobiology, Auburn University, Auburn, AL 36849, USA; zzk0012@auburn.edu; 2 Department of Poultry Science, Auburn University, Auburn, AL 36849, USA

**Keywords:** avian reovirus, transcriptome, RNA-seq, gene expression, cell culture

## Abstract

Avian reovirus (ARV) is an important pathogen of poultry, yet the molecular responses to ARV across cell types remain unknown. The present study explores the differential transcriptomic responses to ARV S1133 infection in three cell types, i.e., chicken embryo kidney (CEK), chicken embryo liver (CELi), and macrophage-derived cells (HD11) at 6, 12, and 24 h post-inoculation (hpi). CELi cells exhibited the highest viral replication rates at all timepoints, with maximal titer observed at 24 hpi, whereas HD11 cells showed limited viral replication but extensive host transcriptional activity. Differential gene expression analysis revealed that macrophage-derived (HD11) cells, despite the lower viral load, presented the most pronounced transcriptional changes. CEK cells demonstrated a unique activation of immune-related pathways, specifically those related to lymphocyte chemotaxis and type II interferon response. CELi cells showed upregulation of expression of genes involved in defense against viruses. Protein–protein interaction (PPI) analysis identified key antiviral genes, including IFI6, OASL, RSAD2, SAMD9L, and MX1, as central nodes. In CELi, significant alternative splicing events were observed in transcripts of several genes, including those implicated in immunity. Taken together, results indicate that inoculation of ARV triggered cell-type and time-dependent viral replication and stimulated transcriptional activity linked with unique but functionally interconnected pathways.

## 1. Introduction

Avian reovirus (ARV) remains relevant as a pathogen of significant interest as it affects poultry worldwide [1], causing substantial economic losses to the poultry industry [2]. The virus can cause various clinical manifestations, such as viral arthritis and tenosynovitis [3], intestinal lesions [4], hepatitis [5,6], pancreatic lesions [4], myocarditis and hydropericardium [7,8], and immunosuppression [9,10].

While the ARV-induced pathogenesis, symptoms, and lesions have been extensively reported and discussed, the transcriptome-wide cellular response, particularly in primary chicken embryo cell cultures, has not been investigated. In primary chicken embryo fibroblasts (CEFs), a direct relationship between autophagy and ARV replication [11], as well as apoptosis following intra-endosomal virion disassembly [12] have been reported. In a secondary fibroblast cell line (DF-1), a sustained antiviral response mediated by interferon-stimulated genes (ISGs) was observed [13].

While the relevance of interferons (IFNs) in the context of ARV infection has been studied, their function remains ambiguous. ARV protein σA-mediated antagonism to IFNs in CEFs [14] and no significant upregulation of IFNs in DF-1 cells [13] have been indicated. Despite the viral protein-mediated resistance, induction of both type-α and type-β IFNs in CEFs [15], as well as in joints of specific-pathogen-free (SPF) chickens [16], has been indicated following infection with ARV. A recent study investigating the transcriptome of spleens of chickens infected with ARV suggested a possible downregulation of IFN-β by overexpression of interleukin-4-induced-1 [17]. Earlier investigations had demonstrated an in vivo induction of IFNs following intratracheal or subcutaneous inoculation of pathogenic or attenuated ARV strains into white leghorn chickens [18]. Later, the same authors reported a lack of induction of IFNs by four virulent ARV strains in chicken kidney (CK) and chicken embryo kidney (CEK) cells [19]. Contrastingly, ultraviolet-inactivated ARV-induced IFNs effectively in these two cell types and attenuated ARV-induced priming-dependent IFN response in aged CEFs [19]. These discrepancies in reports suggest that the ARV-induced transcriptional changes could be cell-type-dependent and warrant a more comprehensive analysis.

The objective of the current study was to investigate the transcriptome-wide changes in gene expression patterns at various timepoints post-inoculation in three different cell types, i.e., primary chicken embryo liver (CELi), CEK cells, and a macrophage-derived continuous cell line (HD-11).

## 2. Materials and Methods

### 2.1. Cell Culture

Chicken embryo kidney and liver cells were prepared from specific pathogen-free eggs at 19 and 15 days of embryonation (DOE), respectively. Briefly, kidneys and livers were collected at respective DOE, minced with scissors, and homogenized in 0.05% Trypsin-EDTA solution (Gibco, Grand Island, NY, USA). The homogenate was filtered with a 40-micron filter, centrifuged at 100× *g* for 10 min, and dissolved in 50 mL of growth medium (composition described below). HD-11 cells were kindly provided by Dr. Li Zhang (Mississippi State University, MS).

All three cell types were cultured in Dulbecco’s Modified Eagle Medium (DMEM) (Corning, Corning, NY, USA) supplemented with 10% fetal bovine serum (FBS) (HyClone, Logan, UT, USA) and 2% penicillin–streptomycin with L-Glutamine (Corning, Corning, NY, USA) and 3% sodium pyruvate (Corning). The cells were maintained at 37 °C in a humidified incubator with 5% CO_2_.

### 2.2. S1133 Inoculum Preparation and Dose Determination

The ARV S1133 strain from a stock prepared previously [20] was passaged once in CELi cells. The harvested cell lysate was freeze–thawed three times to release the virus. The virus was titrated on CELi cells with 8 replicates per dilution and the titer was calculated to be 10^9.33^ 50% tissue culture infective dose (TCID_50_)/mL, using the Reed and Muench method [21]. This supernatant was diluted in DMEM to prepare inoculum at the final dose of 10^6^ TCID_50_/mL.

### 2.3. Virus Inoculation

The cells were grown in one 6-well plate per cell type, for each timepoint and treatment. The growth medium was removed, monolayers were washed with 1 mL of phosphate-buffered saline, and 100 μL of ARV S1133 at 10^6^ TCID^50^/mL was inoculated onto the monolayers of each well. To account for potential expression changes due to culture’s duration or temporal metabolic changes, we included mock controls for each timepoint. Control wells received the same volume of DMEM only. Immediately after inoculation of the virus, 1.5 mL of DMEM containing 2% FBS was added to each well for post-inoculation maintenance. At 6, 12, and 24 h post-inoculation (hpi), each plate to be harvested was removed from the incubator one at a time, placed on ice, and the medium was removed. For immediate RNA release from the cells, 1 mL of RLT lysis buffer (Qiagen, Hilden, Germany) was added to each well. The cells were mixed with RLT using a 21-gauge needle syringe and pipetted into 1.5 mL tubes. The homogenate was frozen immediately at −80 °C until RNA extraction.

### 2.4. Quantitative PCR for Viral Load

RNA was extracted from 5 of the 6 samples using the RNeasy Mini Kit (Qiagen, Hilden, Germany) as per the manufacturer’s instructions. Viral RNA load was quantified by targeting the M1 gene of ARV. Total RNA was denatured for 10 min at 95 °C and RNA was reverse-transcribed using the LunaScript RT SuperMix Kit (New England Biolabs, Ipswich, MA, USA). The qPCR was performed using Forget-Me-Not™ Universal Probe qPCR Master Mix, with the following primers: forward (5′-ATG GCC TMT CTA GCC ACA CCY G-3′), reverse (5′-CAA CGA RAT RGC ATCA ATA GTAC-3′) and probe (5′-TGC TAG GAG TCG GTT CTC GYA-3′) amplifying 80 base pairs between bases 13–93 of the M1 gene [22]. GAPDH gene was targeted to normalize the viral loads, using the primers designed in-house as follows: forward (5′-TGG TGG CCA TCA ATG ATC CC-3′) and reverse (5′-ACC TGC ATC TGC CCA TTT GA-3′), and probe (5′-ACT GTC AAG GCT GAG AAC GG-3’), amplifying the 184 base pairs between bases 83 and 267 of the *Gallus gallus* GAPDH (GenBank accession AF047874.1). All PCRs were conducted using the qTOWER^3^ PCR Thermal Cycler (Analytik Jena, Jena, Germany). Amplification peaks were analyzed by qPCRSoft program version 4.1. Relative viral RNA loads were calculated using the following formula: $\log\left(2^{-(GAPDH\;C_{t}-ARV\;C_{t}\;)}\right)$.

### 2.5. RNA Sequencing

The RNA aliquots were submitted to GENEWIZ (South Plainfield, NJ, USA), where the samples were quantified using a Qubit 2.0 Fluorometer (Life Technologies, Carlsbad, CA, USA), and RNA integrity was checked using Agilent TapeStation 4200 (Agilent Technologies, Palo Alto, CA, USA). ERCC RNA Spike-In Mix (ThermoFisher Scientific, Waltham, MA, USA) was added to normalized total RNA before library preparation following the manufacturer’s protocol.

The RNA sequencing libraries were prepared using the NEBNext Ultra II RNA Library Prep Kit for Illumina, following the manufacturer’s instructions (New England Biolabs, Ipswich, MA, USA). Briefly, mRNAs were initially enriched with Oligod(T) beads. Enriched mRNAs were fragmented by heating for 15 min at 94 °C. First-strand and second-strand cDNA were subsequently synthesized. cDNA fragments were end-repaired and adenylated at 3′ ends, and universal adapters were ligated to cDNA fragments, followed by ligation of oligonucleotides for indexing (barcoding) and library enrichment by PCR with limited cycles. The sequencing libraries were validated on the Agilent TapeStation (Agilent Technologies, Palo Alto, CA, USA) and quantified by using Qubit 2.0 Fluorometer (ThermoFisher Scientific, Waltham, MA, USA) as well as by quantitative PCR (KAPA Biosystems, Wilmington, MA, USA).

The sequencing libraries were clustered on one flow-cell lane. After clustering, the flow cell was loaded on the Illumina instrument (4000 or equivalent) according to the manufacturer’s instructions. The samples were sequenced using a 2 × 150 bp paired-end configuration. Image analysis and base calling were conducted by the Control software. Raw sequence data (.bcl files) generated from the sequencer were converted into fastq files and de-multiplexed using Illumina’s bcl2fastq 2.17 software. One mismatch was allowed for index sequence identification.

### 2.6. Read Processing and Differential Expression Analyses

Raw sequencing reads were processed using fastp program [23], version v0.23.4, for quality control and trimming. Reads were then aligned to the *Gallus gallus* genome (GenBank accession GCA_016699485.1) using HISAT2 [24] version 2.2.1. For quantification, featureCounts [25] version 2.0.1 was used to count the number of reads aligned against the reference. The differential expression analysis was performed on the output of featureCounts [25] subread module version 2.0.1 using edgeR [26] version 4.2.1. The counts of differentially expressed genes (DEGs) were filtered at log2 fold change > 1 and *p* < 0.05 adjusted with Benjamini–Hochberg (BH) correction [27] to account for the false discovery rate. The output was used to create bar plots for the numbers of DEGs identified for each cell type and timepoint. Unique genes across timepoints were pooled and used to generate an UpSet plot [28] using the UpSetR library [29] version 1.4.0. The statistical analyses and plotting were performed using RStudio version 2024.04.2+764 [30,31].

### 2.7. Pathway Annotation and Comparison

The lists of DEGs obtained from edgeR output were used as input to compare pathways enriched in each cell type using Metascape [32], version 3.5.20240901. The pathway networks were visually enhanced using Cytoscape version 3.10.1 [33], and the most significant pathway of the cluster was annotated using AutoAnnotate [34] version 1.3.0.

### 2.8. Protein–Protein Interaction (PPI) Analysis

The interactions of proteins for each cell type were analyzed by inputting the lists of DEGs into the STRING database [35]. A PPI clustering pattern was observed only for CELi cells and is reported herein. The cluster networks were visually enhanced using Cytoscape version 3.10.1 [33].

### 2.9. Isoform Switch Analysis

Quality trimmed reads were aligned using splice-aware mapper STAR [36] version 2.7.11b, followed by transcript assembly with StringTie [37] version 2.2.3. Ballgown-type outputs [38] were generated comprising transcript abundance estimates, and differential isoform expression analysis was performed using IsoformSwitchAnalyzeR [39] version 2.4.0 to predict isoform usage following ARV inoculation.

## 3. Results

### 3.1. Avian Reovirus Replication in Different Cell Types

**Figure 1:**
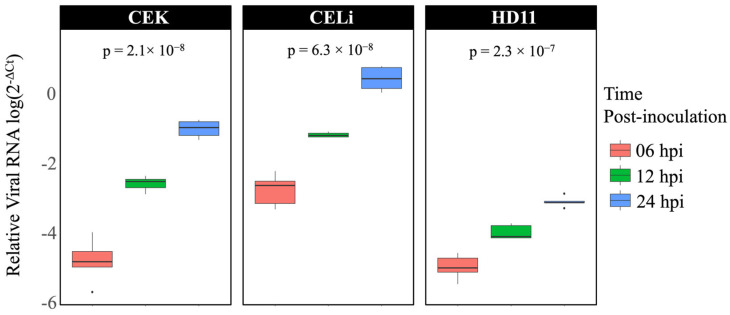
Avian reovirus S1133 replication in various cell types over time (*n* = 5). X-axes indicate the sampling timepoint in hours post-inoculation. Y-axes represent relative viral RNA in terms of $\log\left(2^{-(GAPDH\;C_{t}-ARV\;C_{t}\;)}\right)$ quantified using qPCR targeting ARV M1 gene and normalized against housekeeping gene GAPDH. The exponential increase in viral RNA levels can be appreciated for each cell type, with HD11 cells showing the least efficient replication. The highest viral RNA was detected in chicken embryo liver cells at 24 h post-inoculation.

Avian reovirus S1133 replication was evaluated across the three different cell types, CEK, CELi, and HD11, at multiple timepoints post-inoculation. Figure 1 shows the relative viral RNA levels, quantified using qPCR targeting the ARV M1 gene and normalized against the housekeeping gene GAPDH. The data revealed a time-dependent increase in viral RNA across all cell types, with the highest levels observed in CELi cells at 24 hpi. The replication was greater in CEK cells compared to HD11 cells.

### 3.2. Differential Gene Expression Analysis

**Figure 2:**
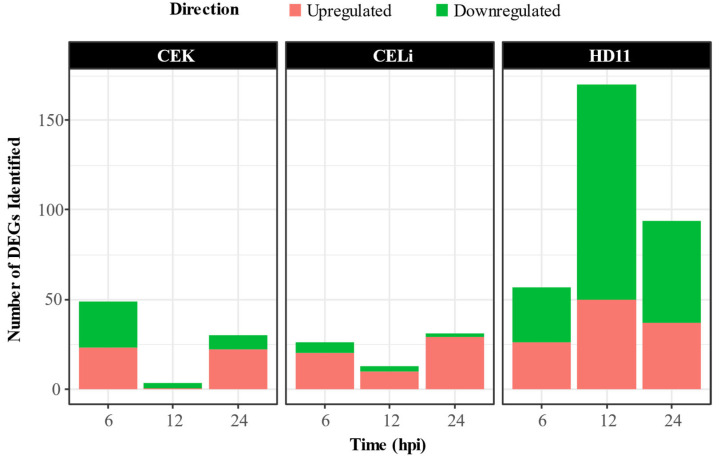
Differentially expressed genes (DEGs) in avian reovirus (ARV) S1133-inoculated versus DMEM-inoculated control cells across timepoints (*n* = 5). Primary chicken embryo kidney (CEK) and liver (CELi) cells and immortalized macrophage-like cells (HD-11) were inoculated with either ARV S1133 or DMEM. In CEK and CELi cells, a decline in the number of DEGs was observed at 12 hpi. The highest number of DEGs was identified in HD-11 cells, especially at 12 hpi.

Differential gene expression compared to uninoculated control cells was assessed across the three cell types infected with ARV S1133 at 6, 12, and 24 hpi. As summarized in the bar plot (Figure 2), CEK cells had 49 DEGs (23 up- and 26 downregulated) at 6 hpi, decreasing to 4 (1 up- and 3 downregulated) DEGs at 12 hpi, before rising again to 30 (22 up- and 8 downregulated) DEGs by 24 hpi. Similarly, CELi cells had 26 (20 up and 6 downregulated), 13 (10 up- and 3 downregulated), and 31 (29 up- and 2 downregulated) DEGs at 6, 12, and 24 hpi, respectively.

**Figure 3:**
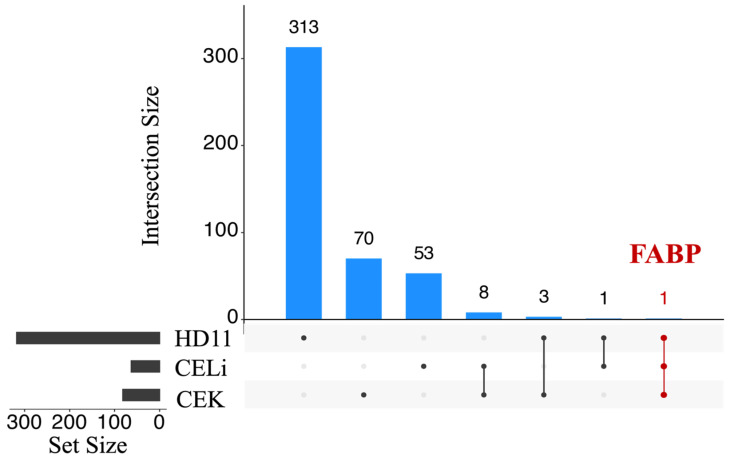
UpSet plot of differentially expressed genes (DEGs) between avian reovirus S1133 infected and control samples in various cell types. The gray bars represent DEGs contributed by samples from CEK, CELi, and HD11 cells with ARV S1133 or DMEM inoculation. The dots connected by lines on the X-axis indicate the cell types for which common DEGs were identified. The numbers above the blue bars represent the number of DEGs shared. Only one common DEG (representing FABP4, intersection colored red) was identified across all three cell types, indicating a divergent transcriptional response.

In contrast, HD11 cells exhibited the highest DEG counts, with 57 (26 up- and 31 downregulated), 170 (50 up- and 120 downregulated), and 94 (37 up- and 57 downregulated) DEGs at 6, 12, and 24 hpi, respectively (Supplementary Data S1). As illustrated in Figure 3, a “total” of 82, 63, and 318 unique DEGs were identified for CEK, CELi, and HD-11, respectively. Only one DEG, i.e., fatty acid-binding protein 4 (FABP4), was found to be common among all three cell types (Figure 3).

### 3.3. Protein–Protein Interaction Network

**Figure 4:**
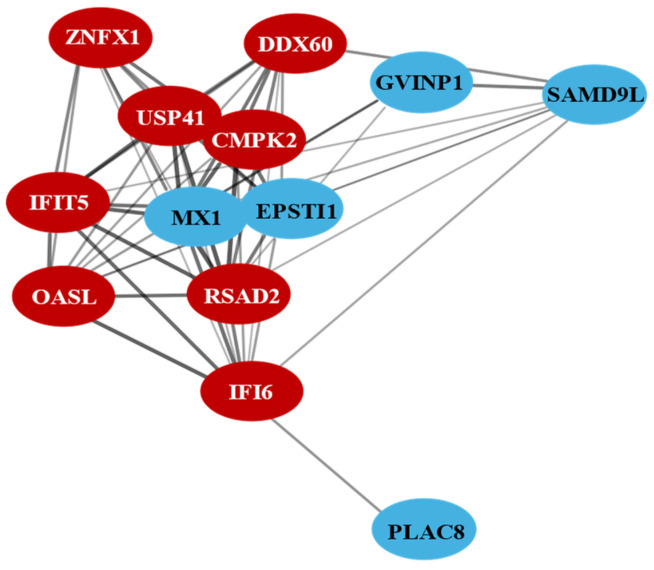
Protein–protein interaction (PPI) network of genes involved in cellular antiviral response (STRING database analysis). Genes differentially expressed between control and S1133-treated chicken embryo liver cells were analyzed to create a protein–protein interaction network. The red color indicates the genes commonly observed in CELi and embryonic liver tissue. The figure includes 2 additional STRING database-expanded interactors, ZNFX1 and PLAC8, beyond the identified CELi 24 hpi DEGs, included to illustrate network context.

A PPI network was constructed to examine the interactions between genes involved in the cellular response to ARV S1133 at various timepoints post-inoculation. The analysis revealed key immune-related genes (Figure 4: interferon alpha-inducible protein 6 (IFI6) associated with inhibition of viral replication by promoting apoptosis; radical S-adenosyl methionine domain-containing 2 (RSAD2, viperin) involved in viral replication inhibition by disrupting lipid rafts necessary for virus budding; myxovirus resistance protein 1 (MX1), a GTPase linked to viral replication inhibition through interferons; and 2′-5′-oligoadenylate synthetase-like (OASL) which is involved in degradation of viral RNA by activating RNase, as key nodes in the network underscoring their importance in mediating the cellular defense against ARV S1133 infection).

### 3.4. Pathway Enrichment: Gene Ontology Analysis

**Figure 5:**
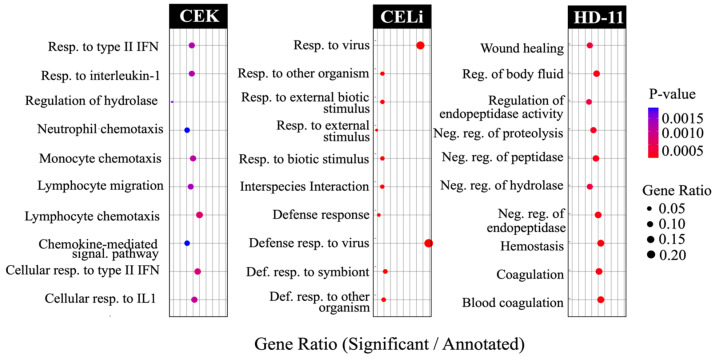
Top 10 pathways enriched in different cell types following avian reovirus inoculation as determined using differentially expressed genes (DEGs). The plots indicate pathways representing gene ontology (GO) database terms enriched in each cell type, i.e., chicken embryo kidney cells (CEK), chicken embryo liver cells (CELi), and macrophage-derived cell line (HD11) following avian reovirus S1133 inoculation. The size of the dots indicates the ratio of differentially expressed genes (DEGs) to the total number of DEGs annotated in the database for a given pathway. The red-to-blue color gradient indicates the distribution of *p*-values from lower to higher, respectively. The GO terms associated with these pathways are provided in Supplementary Data S2.

As illustrated in Figure 5, an analysis of unique DEGs for biological process enrichment indicated a distinct profile of significantly upregulated pathways for each cell type. In CEK cells, immune response-related pathways were significantly enriched, including lymphocyte chemotaxis (GO:0048247, *p* < 0.01) and cellular response to type-II interferon (GO:0071346, *p* < 0.01). Additional processes such as chemokine-mediated signaling and regulation of hydrolase activity were also enriched (*p* < 0.01).

In CELi cells, defense response pathways were strongly represented, with enrichment for defense response to symbiont (GO:0140546, *p* < 0.001) and external biotic stimulus (GO:0043207, *p* < 0.001). Response to virus and interspecies interaction processes were also significantly overrepresented (*p* < 0.001). In HD11 cells, coagulation-related processes were prominent, including blood coagulation (GO:0007596, *p* < 0.01) and negative regulation of endopeptidase activity (GO:0010951, *p* < 0.01). Similar processes like wound healing and regulation of body fluid levels were additionally enriched (*p* < 0.01).

### 3.5. Network Analysis of Pathways

**Figure 6:**
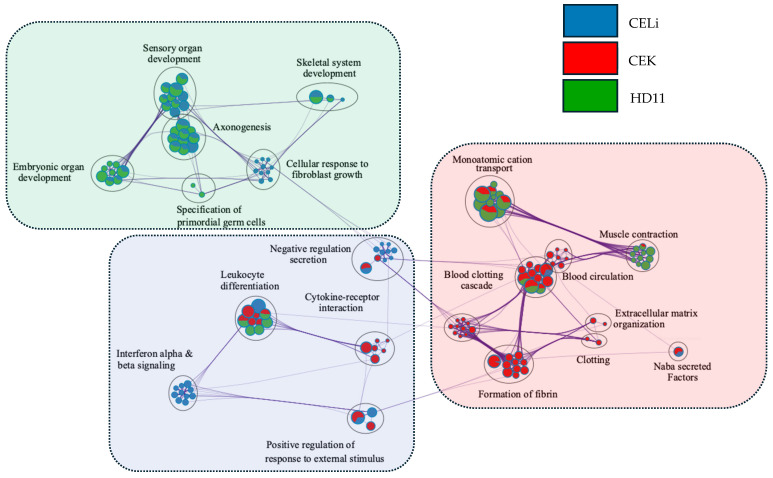
Pathway connectedness network of differentially expressed genes (DEGs) from each cell type. Each circle represents a node, where each node corresponds to a cluster of DEGs (mapped against the human gene ontology database in Metascape). Nodes are linked by edges representing the distance between clusters, which is calculated based on pathway similarity. The size of each node is proportional to the number of DEGs within the cluster. The circles next to each pathway name indicate clusters of closely related pathways, highlighting functionally similar or overlapping biological processes (only the most significant pathway is shown here). The colors of the pies within the charts display the percentage of DEGs derived from each cell type (legend on the top right). The pathway connections indicate connectedness of the cellular response to avian reovirus infection despite a predominantly characteristic functional profile for each cell type (dotted rectangles with respective colors). Gene ontology terms associated with the network analysis as well as the raw network file in .sif format are provided in Supplementary Data S3.

The pathway analysis using DEGs from three cell types revealed distinct but interconnected pathways (Figure 6) that were significantly upregulated in different cell types following infection or treatment. The pie charts at each node indicate the variable percentage of DEGs from each cell type, representing enrichment of a certain pathway.

The clusters primarily associated with CELi cells displayed a significant enrichment in pathways related to viral replication, leukocyte differentiation, and host responses to viral invasion. Genes in this cluster are involved in processes such as viral genome replication, transcriptional regulation by viral proteins, and antiviral immune responses.

DEGs from CEK cells primarily contributed to pathways related to the complement and coagulation cascades. This indicates that these cells had enrichment of processes involved in coagulation and hemostasis, likely in response to inflammation or cell damage induced by infection. Pathways such as platelet degranulation and the response to elevated platelet cytosolic calcium levels were also prominent.

Interestingly, DEGs in HD11 cells contributed to pathways predominantly related to growth and metabolism. The pathways included axonogenesis and sensory organ development, among others.

### 3.6. Isoform Switching Analysis

**Figure 7:**
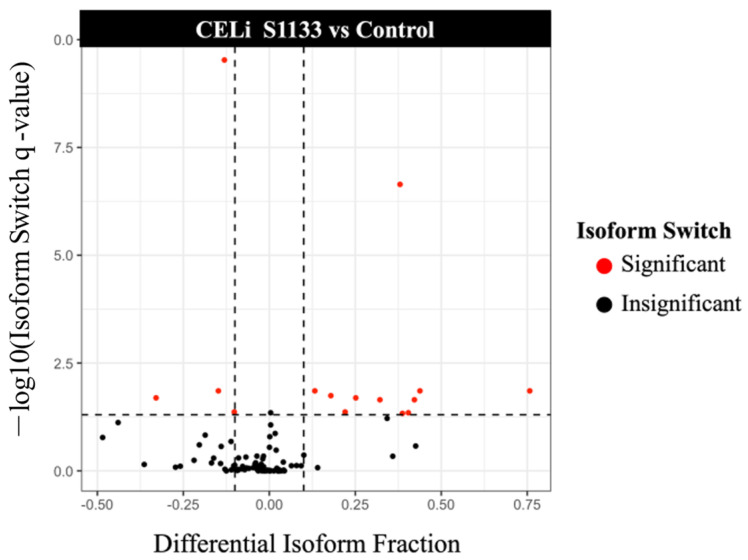
Volcano plot depicting splicing isoform switch in chicken embryo liver cells (CELi) following ARV S1133 inoculation. Volcano plot highlighting the significant isoform switching between control and S1133-treated samples. Differential isoform fraction on the X-axes represents the difference between ratios of isoform expression to total gene expression for control and S1133-inoculated samples. The red dots represent significant isoform switches with substantial changes in isoform fraction between control and S1133-infected samples. Those on the right or left show increase or decrease in overall isoform switch, respectively.

Since CELi indicated upregulation of pathways related to defense against the virus, splicing isoform usage was investigated in the infected CELi cells. As shown in the volcano plot (Figure 7), significant isoform switches for transcripts from 16 genes (shown as red dots) were observed.

**Figure 8:**
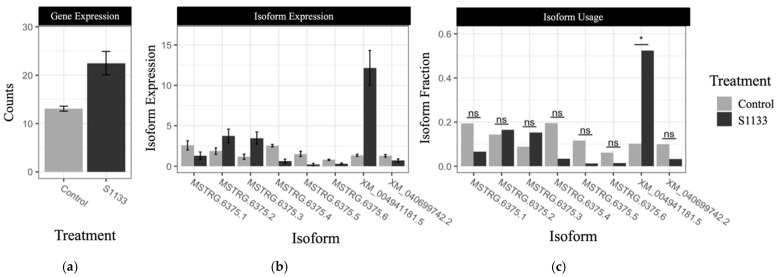
Visualization of the splicing isoform differences for the ANKRD17 gene between control and S1133-treated samples. (**a**) The expression of the ANKRD17 gene is shown. (**b**) Bar plots show counts of isoform transcripts for each treatment. (**c**) Bar plots show the differential usage of the expressed isoforms isoform fraction. A significant (an asterisk (*) denotes significance) increase in isoform XM_040694181.5 usage in S1133-inoculated groups was observed, suggesting differential alternative splicing events upon ARV infection.

Most of the genes showing changes in alternative RNA splicing patterns have been associated with cancer. One gene, ANKRD17, has been previously linked with antiviral immunity through RIG-1-like receptor-mediated signaling. A notable switch in ANKRD17 isoform usage between control and ARV S1133-infected samples has been illustrated in Figure 8, where isoform XM_040694181.5 showed a substantial increase in expression following infection. The top 10 genes exhibiting significant isoform switch based on q-value and their functions as well as the information on those 16 significant isoform switch values are shown in Supplementary Data S4.

## 4. Discussion

The study aimed to explore ARV-induced transcriptomic changes across three cell types: CEK, CELi, and HD11 at 6, 12, and 24 hpi. We chose CEK cells because of their high permissiveness of replication to ARV infections in vitro [18], despite absence of characteristic lesions in vivo. The CELi was chosen because ARV-induced perihepatitis [5,6] has not yet been investigated in vitro. The primary CEK and CELi cultures were selected because of their pathological relevance and heterogeneous cell populations [40,41], as they better represent gene expression patterns of a tissue compared to secondary cell lines with more homogeneous cell populations. The cell line HD11 was selected to ascertain the immune responses of macrophages due to their relevance in ARV dissemination across tissues [42], potential association with impaired phagocytosis [43], and immunosuppression [44].

The quantification of viral RNA levels at various timepoints demonstrated a cell-type-dependent replication efficiency of ARV S1133, with the highest viral loads observed in CELi cells, followed by CEK cells, and the least efficient replication in HD11 cells. A higher replication of ARV in CELi compared to CEK is concordant with the previous findings on differences in the sensitivity of these two cell types to ARV infection [45]. Moreover, these results corroborate the observations of lower ARV replication in kidneys compared to livers in vivo and hence, a lesser induction of lesions in the former [45]. Conversely, a higher replication efficiency of infectious bronchitis virus (IBV) in CEK compared to CELi has been demonstrated previously [46], further affirming that replication efficiency of a virus in cultured cells could reflect its tissue tropism in vivo.

While ARV S1133 has been shown to replicate in peripheral blood monocyte cultures [47], and chicken bone marrow-derived macrophages, as efficiently as in CK cells [48], a lower replication of S1133 in HD11 cells in our study showed a restricted efficiency of productive ARV infection in this secondary cell line [49]. Interestingly, despite exhibiting limited ARV replication, macrophage-like cell line had the highest number of DEGs at all timepoints. Such a negative correlation of viral loads with number of DEGs has been reported for swine macrophages infected with the African swine fever virus [50]. Contrastingly, this negative correlation of viral replication with host gene expression remains at odds with the observations on IBV-infected HD11 cells [51]. Since our approach included viral genomic RNA as well as mRNA quantification, the viral loads represent both viral genomic RNA copies as well as the viral mRNA, unlike minus-strand-specific PCRs, which signify progeny virus genome replication.

Interestingly, a lower number of DEGs was observed at 12 hpi for CEK and CELi, with a subsequent increase again at 24 hpi. The balance between viral replication and host response in these cells could be a potential viral strategy to maintain a favorable environment for propagation while avoiding overwhelming host shutoff. Neither CEK nor HD11 cells had any common DEGs across timepoints. However, three genes, i.e., OASL, IFIT5, and IFI6, were consistently differentially regulated in CELi cells at all three timepoints tested. These interferon-stimulated genes (ISGs), along with others, were also part of the protein–protein interaction network generated using DEGs expressed in CELi cells at all timepoints. These genes appear to be a part of the core antiviral response against avian reovirus in vivo [16,17], in ovo [52], and in vitro [13], as well as against other viruses [53]. The role of the pro-inflammatory ISGs in CELi cells could contribute to perihepatitis observed during in vivo infections. Remarkably, despite the most efficient ARV replication in CELi cells, this cell type had the lowest count of DEGs. CELi cells exhibited the highest viral replication likely due to their permissiveness and metabolic support for ARV, aligning with in vivo findings of liver tropism [5,6]. In contrast, HD11 cells, despite limited replication, showed robust transcriptional responses, suggesting active sensing and restriction of viral replication, a characteristic of macrophages.

In the ARV-infected CEK cells, pathways pertaining to type-II IFNs and IL-1 were enriched, validating the findings of previous researchers [15,16]. This is in contrast with previous studies demonstrating ineffective induction of IFNs in CEK cells [19] and the downregulation of IFN-β in spleens of chickens infected with ARV [17]. The enrichment of pathways related to heterophil and lymphocyte chemotaxis and migration in CEK cells suggest chemokine-induced preferential recruitment of these immune cells [54]. Our findings confirm the observations made by Ni and Kemp in the in vivo study [55], where foci of heterophil and lymphocytic infiltration were found despite absence of apparent lesions. It can be speculated that these cells might be recruited during ARV infection in vivo, resulting in neutralization of the virus, whereas the susceptibility of the cultured CEK cells could be explained by their isolated state.

Surprisingly, HD11 cells exhibited differential regulation of individual pathways associated with blood such as fibrinogenesis and complement cascade activation. Similar observations have been made with IBV-infected chicken kidneys, where DEGs related to complement factors and blood vessel-associated pathways were identified [56]. Moreover, the role of the complement system in immune responses has been described for the influenza virus [57], human immunodeficiency virus [58], Sindbis virus [59], and dengue virus [60] infections. Additionally, an enrichment in several pathways involved in the regulation of endopeptidase activity could be speculatively associated with reovirus replication. Cathepsins, a class of endopeptidases involved in lysosomal proteolysis, have been linked to reovirus entry and disassembly [61]. Since the outer capsid processing of various reoviruses in macrophage-like cells has been associated with endopeptidase cathepsin-S [62], the regulation of endopeptidase activity in ARV-infected HD11 cells suggests a potential cellular response to infection to prevent ARV entry and disassembly. These results contradict in vivo studies with other viruses where significantly increased endopeptidase activity was observed in the pancreas of reovirus type-3-infected suckling mice [63] and high endopeptidase expression was related to severe outcomes with SARS-CoV-2 infection [64].

The identification of fatty acid-binding protein 4 (FABP4) as the only common DEG among all three cell types is a novel finding in the context of ARV infection. While FABP4 has not been studied in the context of reovirus-induced arthritis or tenosynovitis, it has been described as a biomarker of human knee osteoarthritis, where patients exhibited significantly higher systemic and synovial FABP4 [65]. Interestingly, a pro-inflammatory role of FABP4 has been described in the pathogenesis of chronic tendinopathy in humans. The results were confirmed with experimental tendon degeneration in mice [64]. Moreover, a higher level of FABP4 in lungs and circulation of patients infected with SARS-CoV-2 and an experimental alleviation of viral loads, lung damage, and fibrosis in infected hamsters treated with FABP4 inhibitors underscored its importance as a biomarker [66].

Effects on alternative splicing of host precursor mRNA, resulting in expression of different transcript isoforms and their ultimate translation into proteins with divergent functions, have been reported previously for a mammalian orthoreovirus [67], as well as for influenza viruses [68]. In the present study, a novel observation of significant isoform switches due to alternative splicing of host transcripts in infected CELi cells adds another layer of complexity to the current understanding of the host response against ARV. A notable switch in isoform of the ANKRD17 gene transcript was especially interesting, given the implication of the gene product in antiviral immune responses via the retinoic acid-inducible gene-I (RIG-I)-like receptor (RLR) signaling pathway in influenza virus infections [69], and pro-inflammatory responses in bacterial infections [70]. The human orthologs of some other DEGs exhibiting isoform switches in ARV-infected cells are associated with cellular response to cancer. Since the oncolytic potential of ARVs has been documented previously [71,72,73], the preliminary observation on differential isoform usage among various cancer-linked genes in the infected host cells warrants further exploration of ARV-induced alternative splicing.

In conclusion, the study describes a detailed molecular snapshot of ARV infection in three cell types, where viral replication efficiency, host gene expression, and cellular responses remain cell-type-specific but somewhat interconnected and uniquely meaningful. Several hypotheses on molecular changes following ARV inoculation were generated in the study and further studies validating the findings on protein translation, protein–protein interactions, and alternative splicing are warranted.

## Data Availability

The raw reads are available at Sequence Read Archive, National Center for Biotechnology Information (NCBI) under the accession number PRJNA1102662.

## Supplementary Materials

The following supporting information can be downloaded at https://www.mdpi.com/article/10.3390/v17111489/s1, Data S1: complete list of DEGs; Data S2: gene ontology (GO) terms associated with pathways presented in Figure 5; Data S3: GO terms associated with the network analysis in Figure 6 and raw network file; Data S4: genes indicating isoform switching and isoforms.
